# A survey of the awareness and knowledge of oral cancer among residents in Beijing

**DOI:** 10.1186/s12903-022-02398-6

**Published:** 2022-08-28

**Authors:** Xing-Hong Zhou, Ying Huang, Chao Yuan, Shu-Guo Zheng, Jian-Guo Zhang, Xiao-Ming Lv, Jie Zhang

**Affiliations:** 1 Department of Oral and Maxillofacial Surgery, Peking University School and Hospital of Stomatology, National Clinical Research Center for Oral Diseases, National Engineering Laboratory for Digital and Material Technology of StomatologyBeijing Key Laboratory of Digital Stomatology, Beijing, 100081 China; 2 Department of Preventive Dentistry, Peking University School and Hospital of Stomatology, National Clinical Research Center for Oral Diseases, National Engineering Laboratory for Digital and Material Technology of Stomatology, Beijing Key Laboratory of Digital Stomatology, Beijing, 100081 China

**Keywords:** Oral cancer, Beijing, Questionnaire survey, Awareness

## Abstract

**Background:**

The present study aimed to investigate oral cancer awareness and its related knowledge among residents in Beijing.

**Methods:**

A questionnaire survey was conducted among Beijing residents concerning their knowledge of oral cancer, and its prevention and treatment.

**Results:**

A total of 3055 questionnaires were completed, 45.8% by males and 54.2% by females. The ages of the respondents ranged from 15 to 93 years; 12.4% were smokers, 1.1% chewed betel nuts, and 82.5% brushed their teeth at least twice a day. Lung cancer was heard of by the most respondents, followed by gastric cancer and liver cancer; oral cancer was the least heard of. More than 60% of respondents were unaware of the risk factors and early signs of oral cancer.

**Conclusions:**

This survey demonstrated a general lack of public awareness and knowledge about oral cancer. Specific measures should be taken to improve public awareness of oral cancer and its prevention and treatment.

## Background

Oral cancer is a common malignant tumour among head and neck cancers. About 355,000 new cases and 177,000 cancer deaths were estimated to have occurred in 2018 worldwide [1]. Oral cancer is becoming a serious global problem. It was estimated that about 48,100 oral and pharynx cancer cases and 22,100 cancer deaths occurred in China in 2015 [2]. Oral cavity is easily accessible for examination, and once abnormalities occur, it should be easy to diagnose, because it affects eating, chewing, pronunciation, and speech, etc. However, more than half of cases were diagnosed with clinically advanced oral cancer. There are a variety of explanations for the low early diagnosis rate and the advanced stage of most oral cancers at presentation, such as a lack of public attention to oral health, a lack of awareness of early symptoms and potential malignant lesions of oral cancer, and bad or unhealthy lifestyles [3]. Therefore, increased public awareness of oral cancer, its risk factors, and early signs, will reduce people’s exposure to risk factors or will prompt them to seek medical attention if they find potential malignant or early-stage lesions. This would help improve the survival rate of patients with oral cancer, improve patients' quality of life after surgery, and reduce the consumption of medical resources.

Unfortunately, there is lack of studies reporting oral cancer knowledge of people from China. Therefore, the aim of the present study was to determine the current status of oral cancer and its related knowledge among Beijing residents using a questionnaire-based survey.

## Methods

### Respondents

The respondents were non-medically related permanent residents of Beijing, who were not doctors, nurses, or medical students. A permanent resident was defined as a person who has lived in Beijing for more than half a year.

### Sampling method

The sampling method was convenience sampling. Investigators conducted surveys in commercial centers, parks and communities with relatively large flow of people and relatively complete structures in various districts of Beijing, as long as passers-by voluntarily participated and met the conditions.

### Questionnaire design

Based on previous similar studies and their contents about the treatment of oral cancer, a questionnaire was designed. It mainly included the following three aspects: (i) general situation: gender, age, occupation, educational background, residence, family annual per capita income, marital status, and medical insurance; (ii) lifestyle and habits: whether they smoked, drank alcohol, chewed betel nuts, the frequency of tooth brushing and mouth self-examination, and the frequency of visiting a doctor of stomatology; and (iii) oral cancer-related knowledge: the awareness of the top ten cancers; whether they had heard of oral cancer and the way; the most common site of oral cancer; the age of onset; whether is preventable or infectious; whether the risk increases with age; the risk factors; possible early presentation; how deal with these symptoms if they occur; the effectiveness of treatment; the cost; mortality rates; whether early detection improves the treatment success rate; and whether changes in lifestyle or habits can reduce the risk of oral cancer.

### Pilot survey

Seven investigators were recruited from among stomatology students, who received unified training and passed a Kappa consistency test (Kappa > 0.9). Face-to-face communication was adopted to complete the questionnaire, which was filled in by the investigator. Respondents with a higher level of education could fill in the form themselves. The investigators answered the questions raised by the respondents and checked whether the contents are qualified. If there was doubt, the investigator will inquire, confirm, and fill in the form again.

### Statistical methods

SPSS 24.0 statistical software was used (IBM Corp., Armonk, NY, USA). The answers to each question in the questionnaire are expressed in terms of frequency (percentage). In the univariate analysis, a chi-squared test was used to analyse possible associations between general information, lifestyle, and habits, and oral cancer-related knowledge. Items with statistical significance in the single factor analysis were analysed using logistic regression analysis. The odds ratio (OR) and the 95% confidence interval (CI) were calculated. *P* < 0.05 was considered statistically significant.

## Results

### Basic information

The survey lasted 17 months, from May 2018 to September 2019, during which 3055 valid questionnaires were collected. Table 1 shows the demographic characteristics of the respondents.

**Table 1:** Socio-demographic characteristics

Variable	n (%)
Gender	
Male	1400 (45.8)
Female	1655 (54.2)
Age	
15–29 years	1618 (53.0)
30–44 years	963 (31.5)
45–59 years	302 (9.9)
≥ 60 years	172 (5.6)
Education	
Primary or below	60 (1.9)
Middle high	152 (5.0)
High or technical school	344 (11.3)
University or college	1842 (60.3)
Graduate or higher	657 (21.5)
Residence	
Downtown	481 (15.7)
Suburbs	1534 (50.2)
Outer suburbs	923 (30.2)
Rural	117 (3.8)
Marital status	
Married	1340 (43.9)
Single	1642 (53.7)
Widow/Divorced/Separated	73 (2.4)
Income	
< 20,000 Yuan	1135 (37.2)
20,000–40,000 Yuan	597 (19.5)
40,000–60,000 Yuan	493 (16.1)
> 60,000 Yuan	830 (27.2)
Medical insurance	
Yes	2775 (90.8)
No	280 (9.2)

### Lifestyles and habits

Among the respondents, 12.4% were smokers, 1.1% chewed betel nuts, and 82.5% brushed their teeth at least twice a day, whereas 16.0% only brushed once a day. Most of the respondents did not practice oral self-examination: 37.5% of the respondents checked their oral condition only when there were problems, such as ulcers. Of the 1,025 individuals, 62.4% visited the Department of Stomatology less than once a year.

### Awareness rate of cancers

**Fig. 1:**
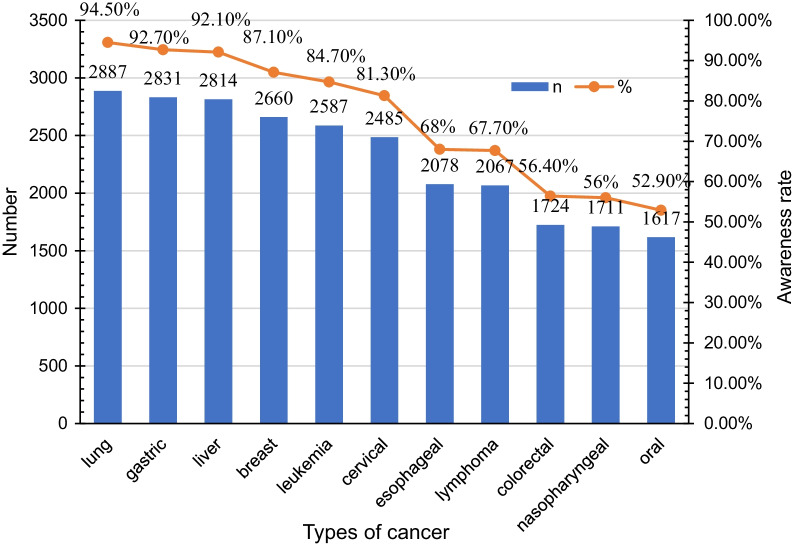
Awareness rate of cancers

Lung cancer was the most heard of cancer (94.5%), followed by gastric cancer (92.7%) and liver cancer (92.1%), while oral cancer was the least recognised (52.9%) (Fig. 1).

Education level (*P* = 0.006), residence (*P* = 0.001), per capita household income (*P* = 0.001), betel nut chewing (*P* = 0.008), frequency of tooth brushing (*P* = 0.001), and frequency of mouth self-examination (*P* = 0.010) were associated significantly with oral cancer awareness (Table 2). In the multivariate analysis, only residence, betel nut chewing, and frequency of mouth self-examination remained statistically significant (Table 3).

**Table 2:** Univariate analysis of factors associated with awareness rate, risk factors, and early signs of oral cancer n (%)

Variable	n	Aware-ness rate	Risk factors				Early signs		
			Age	Smoking	Chewing betel nut	Drinking	Ulcer	White plaques	Red plaques
Total	3055	1617	854	1209	586	779	1222	694	536
		(52.9)	(28.0)	(39.6)	(41.9)	(25.5)	(40.0)	(22.7)	(17.5)
Gender									
Male	1400	726	414	543	586	337	539	290	228
		(51.9)	(29.6)	(38.8)	(41.9)	(24.1)	(38.5)	(20.7)	(16.3)
Female	1655	891	440	666	663	442	683	404	308
		(53.8)	(26.6)	(40.2)	(40.1)	(26.7)	(41.3)	(24.4)	(18.6)
*P*		0.275	0.163	0.333	0.315	0.067	0.297	**0.031**	0.227
Age									
15–29 years	1618	883	468	675	727	426	685	368	301
		(54.6)	(28.9)	(41.7)	(44.9)	(26.3)	(42.3)	(22.7)	(18.6)
30–44 years	963	493	283	398	392	259	387	236	173
		(51.2)	(29.4)	(41.3)	(40.7)	(26.9)	(40.2)	(24.5)	(18.0)
45–59 years	302	148	67	84	89	60	103	60	37
		(49.0)	(22.2)	(27.8)	(29.5)	(19.9)	(34.1)	(19.9)	(12.3)
≥ 60 years	172	93	36	52	41	34	47	30	25
		(54.1)	(20.9)	(30.2)	(23.8)	(43.9)	(27.3)	(17.4)	(14.5)
*P*		0.181	**0.005**	**< 0.001**	**< 0.001**	**< 0.001**	**< 0.001**	**0.037**	0.089
Education									
Primary or below	60	29	12	15	15	11	12	12	12
		(48.3)	(20.0)	(25.0)	(25.0)	(18.3)	(20.0)	(20.0)	(20.0)
Middle high	152	60	18	33	29	21	42	23	19
		(39.5)	(11.8)	(21.7)	(19.1)	(13.8)	(27.6)	(15.1)	(12.5)
High or technical school	344	172	73	114	118	76	110	67	50
		(50.0)	(21.2)	(33.1)	(34.3)	(22.1)	(32.0)	(19.5)	(14.5)
University or college	1842	997	548	755	783	485	766	404	324
		(54.1)	(29.8)	(41.0)	(42.5)	(26.3)	(41.6)	(21.9)	(17.6)
Graduate or higher	657	359	203	292	304	186	292	188	131
		(54.6)	(30.9)	(44.4)	(46.3)	(28.3)	(44.4)	(28.6)	(19.9)
*P*		**0.006**	**< 0.001**	**< 0.001**	**< 0.001**	**< 0.001**	**< 0.001**	**0.001**	0.230
Residence									
Downtown	481	271	141	192	197	138	195	122	92
		(56.3)	(29.3)	(39.9)	(41.0)	(28.7)	(40.5)	(25.4)	(19.1)
Suburbs	1534	848	470	648	690	397	654	367	293
		(55.3)	(30.6)	(42.2)	(45.0)	(25.9)	(42.6)	(23.9)	(19.1)
Outer suburbs	923	444	225	330	328	221	334	178	134
		(48.1)	(24.4)	(35.8)	(35.5)	(23.9)	(36.2)	(19.3)	(14.5)
Rural	117	54	18	39	34	23	39	27	17
		(46.2)	(15.4)	(33.3)	(29.1)	(19.7)	(33.3)	(23.1)	(14.5)
*P*		**0.001**	**< 0.001**	**0.009**	**< 0.001**	**0.001**	**< 0.001**	**0.003**	**0.033**
Marital status									
Married	1340	687	368	503	490	337	512	299	221
		(51.3)	(27.5)	(37.5)	(36.6)	(25.1)	(38.2)	(22.3)	(16.5)
Single	1642	891	467	681	729	423	681	380	302
		(54.3)	(28.4)	(41.5)	(44.4)	(25.8)	(41.5)	(23.1)	(18.4)
Widow/divorced/separated	73	39	19	25	30	19	29	15	13
		(53.4)	(26.0)	(34.2)	(41.1)	(26.0)	(39.7)	(20.5)	(17.8)
*P*		0.264	0.502	0.061	**0.003**	**0.004**	0.487	0.777	0.714
Income									
**< **20,000 Yuan	1135	585	293	422	435	291	430	235	178
		(51.5)	(25.8)	(37.2)	(38.3)	(25.6)	(37.9)	(20.7)	(15.7)
20,000–40,000 Yuan	597	287	147	218	216	129	213	124	92
		(48.1)	(24.6)	(36.5)	(36.2)	(21.6)	(35.7)	(20.8)	(15.4)
40,000–60,000 Yuan	493	259	141	197	201	120	193	114	93
		(52.5)	(28.6)	(40.0)	(40.8)	(24.3)	(39.1)	(23.1)	(18.9)
> 60,000 Yuan	830	486	273	372	397	239	386	221	173
		(58.6)	(32.9)	(44.8)	(47.8)	(28.8)	(46.5)	(26.6)	(20.8)
*P*		**0.001**	**0.004**	**0.003**	**< 0.001**	0.085	**< 0.001**	**0.021**	**0.024**
Medical insurance									
Yes	2775	1477	792	1109	1141	712	1120	639	487
		(53.2)	(28.5)	(40.0)	(41.1)	(25.7)	(40.4)	(23.0)	(17.5)
No	280	140	62	100	108	67	102	55	49
		(50.0)	(22.1)	(35.7)	(38.6)	(23.9)	(36.4)	(19.6)	(17.5)
*P*		0.303	**0.049**	0.451	0.349	0.540	0.302	0.307	0.458
Smoking									
No	2676	1406	745	1075	1104	693	1081	618	475
		(52.5)	(27.8)	(40.2)	(41.3)	(25.9)	(40.4)	(23.1)	(17.8)
Yes	379	211	109	134	145	86	141	76	61
		(55.7)	(28.8)	(35.4)	(38.3)	(22.7)	(37.2)	(20.1)	(16.1)
*P*		0.253	0.881	0.095	0.704	0.602	0.494	0.413	0.176
Chewing betel nut									
No	3022	1592	840	1191	1233	766	1204	685	529
		(52.7)	(27.8)	(39.4)	(40.8)	(25.3)	(39.8)	(22.7)	(17.5)
Yes	33	25	14	18	16	13	18	9	7
		(75.8)	(42.4)	(54.5)	(13.5)	(39.4)	(54.5)	(27.3)	(21.2)
*P*		**0.008**	0.166	0.117	**< 0.001**	0.252	0.189	0.497	0.664
Frequency of tooth brushing									
Zero	45	24	7	14	16	14	15	12	9
		(53.3)	(15.6)	(31.1)	(35.6)	(31.1)	(33.3)	(26.7)	(20.0)
Once in the morning	383	167	80	117	123	69	124	59	46
		(43.6)	(20.9)	(30.5)	(32.1)	(18.0)	(32.4)	(15.4)	(12.0)
Once in the evening	107	56	34	45	41	28	42	22	18
		(52.3)	(31.8)	(42.1)	(38.3)	(26.2)	(39.3)	(20.6)	(16.8)
Twice a day	2416	1305	698	983	1,017	634	987	568	432
		(54.0)	(28.9)	(40.7)	(42.1)	(26.2)	(40.9)	(23.5)	(17.9)
Over three times	104	65	35	50	52	34	54	33	31
		(62.5)	(33.7)	(48.1)	(50.0)	(32.7)	(51.9)	(31.7)	(29.8)
*P*		**0.001**	**0.035**	**< 0.001**	**< 0.001**	**0.020**	**0.005**	**0.010**	**0.007**
Mouth self-examination									
Never	482	240	113	173	182	121	170	91	69
		(49.8)	(23.4)	(35.9)	(37.8)	(25.1)	(35.3)	(18.9)	(14.3)
Nearly every day	435	255	147	191	196	124	199	121	104
		(58.6)	(33.8)	(43.9)	(45.1)	(28.5)	(45.7)	(27.8)	(23.9)
Three or four times a week	991	542	302	411	418	264	417	260	173
		(54.7)	(30.5)	(41.5)	(42.2)	(26.6)	(42.1)	(26.2)	(17.5)
Occasionally	1147	580	292	434	453	270	436	222	190
		(50.6)	(25.5)	(37.8)	(39.5)	(23.5)	(38.0)	(19.4)	(16.6)
*P*		**0.010**	**0.004**	0.323	0.128	0.075	**0.015**	**< 0.001**	**0.001**

*P* value < 0.05 are shown in bold

**Table 3:** Multiple logistic regression analysis of factors associated with awareness of oral cancer

Variable	n (%)	OR	95% CI	*P*
Residence				
Downtown	271 (56.3)	1		
Suburbs	848 (55.3)	0.952	0.772–1.175	0.648
Outer suburbs	444 (48.1)	0.753	0.599–0.946	**0.015**
Rural	54 (46.2)	0.933	0.606–1.437	0.752
Chewing betel nut				
No	1592 (52.7)	1		
Yes	25 (75.8)	2.931	1.298–6.620	**0.010**
Mouth self-examination				
Never	240 (49.8)	1		
Nearly every day	255 (58.6)	1.322	1.013–1.725	**0.040**
Three or four times a week	542 (54.7)	1.182	0.946–1.477	0.142
Occasionally	580 (50.6)	1.031	0.830–1.282	0.783

*P* value < 0.05 are shown in bold

### Source of information about oral cancer

Most of the respondents learned about oral cancer from TV programs (25.2%), phone news (20.3%), WeChat (13.0%), and talking to friends or neighbours (15.5%). Among the respondents, 3.4% had a relative or friend with oral cancer.

### Awareness of the risk factors associated with oral cancer

Approximately 28.0% of the respondents believed that the risk of oral cancer increased with age, and 66.5% did not know whether age was a risk factor for oral cancer. Moreover, 39.6% believed that smoking increased the risk of oral cancer and 55.3% did not know. Tobacco chewing was considered a risk factor of oral cancer by 37.2% of the respondents, whereas 40.9% knew that betel nut chewing was a risk factor, and 56.8% did not know. Only 25.5% respondents believed that those who drank too much alcohol were more likely to develop oral cancer, 61.6% did not know, 10.0% believed that the two were unrelated, and a minority (2.9%) believed that those who drank too much were less likely to develop oral cancer.

Recognition of age as a risk factor was associated with age (*P* = 0.005), education level (*P* < 0.001), residence (*P* < 0.001), per capita household income (*P* = 0.004), medical insurance (*P* = 0.049), frequency of tooth brushing (*P* = 0.035), and frequency of mouth self-examination (*P* = 0.004) (Table 2). In the multivariate analysis, only residence and frequency of mouth self-examination were statistically significant. Those living in the downtown area were more aware that age was a risk factor of oral cancer than those living in outer urban areas, and those who had practiced mouth self-examination were more aware that age was a risk factor of oral cancer than those who did not (Table 4).

**Table 4:** Multiple Logistic regression analysis of factors associated with risk factors of oral cancer

Variable	n (%)	OR	95% CI	*P*
*Age*				
Residence				
Downtown	141 (29.3)	1		
Suburbs	470 (30.6)	0.974	0.771–1.231	0.825
Outer suburbs	225 (24.4)	0.764	0.589–0.992	**0.043**
Rural	18 (15.4)	0.624	0.352–1.106	0.106
Mouth self-examination				
Never	113 (23.4)	1		
Nearly every day	147 (33.8)	1.524	1.130–2.056	**0.006**
Three or four times a week	302 (30.5)	1.310	1.011–1.697	**0.041**
Occasionally	292 (25.5)	1.046	0.809–1.353	0.730
*Smoking*				
Age				
15–29 years	675 (41.7)	1		
30–44 years	398 (41.3)	0.873	0.733–1.039	0.125
45–59 years	84 (27.8)	0.595	0.441–0.804	**0.001**
≥ 60 years	52 (30.2)	0.739	0.485–1.125	0.159
Income				
** < **20,000 Yuan	422 (37.2)	1		
20,000–40,000 Yuan	218 (36.5)	0.977	0.789–1.210	0.829
40,000–60,000 Yuan	197 (40.0)	1.100	0.878–1.379	0.409
> 60,000 Yuan	372 (44.8)	1.242	1.021–1.511	**0.030**
Chewing betel nut				
Age				
15–29 years	727 (44.9)	1		
30–44 years	392 (40.7)	0.761	0.604–0.960	**0.021**
45–59 years	89 (29.5)	0.556	0.391–0.789	**0.001**
≥ 60 years	41 (23.8)	0.413	0.255–0.669	**< 0.001**
Residence				
Downtown	197 (41.0)	1		
Suburbs	690 (45.0)	1.050	0.845–1.305	0.661
Outer suburbs	328 (35.5)	0.767	0.604–0.974	**0.030**
Rural	34 (29.1)	0.881	0.548–1.418	0.603
Marital status				
Married	729 (44.4)	1		
Single	490 (36.6)	0.979	0.779–1.232	0.858
Widow/Divorced/Separated	30 (41.1)	1.825	1.053–3.161	**0.032**
Income				
< 20,000 Yuan	435 (38.3)	1		
20,000–40,000 Yuan	216 (36.2)	0.942	0.761–1.166	0.582
40,000–60,000 Yuan	201 (40.8)	1.116	0.891–1.398	0.338
> 60,000 Yuan	397 (47.8)	1.331	1.096–1.616	**0.004**
*Drinking*				
Age				
15–29 years	426 (26.3)	1		
30–44 years	259 (26.9)	0.836	0.644–1.085	0.177
45–59 years	60 (19.9)	0.627	0.421–0.934	**0.022**
≥ 60 years	34 (43.9)	0.677	0.400–1.146	0.146
Residence				
Downtown	138 (28.7)	1		
Suburbs	397 (25.9)	0.837	0.658–1.066	0.149
Outer suburbs	221 (23.9)	0.743	0.570–0.967	**0.027**
Rural	23 (19.7)	0.758	0.444–1.296	0.312

*P* value < 0.05 are shown in bold

The respondents' perception of smoking as a risk factor for oral cancer correlated with age (*P* < 0.001), education (*P* < 0.001), residence (*P* = 0.009), income (*P* = 0.003), and frequency of tooth brushing (*P* < 0.001) (Table 2). When these factors were included in multivariate analysis, only age and income had statistical significance. People aged 15–29 were more aware of the harmful effects of smoking than people aged 45–59, and those with per capita annual income of over 60,000 yuan were more knowledgeable about the risk (Table 4).

Recognition of chewing betel nuts as a risk factor was associated with age (*P* < 0.001), education (*P* < 0.001), residence (*P* < 0.001), marital status (*P* = 0.003), income (*P* < 0.001), chewing betel nuts (P < 0.001), and frequency of tooth brushing (*P* < 0.001) (Table 2). In the multivariate analysis, age, residence, marital status, and income were statistically significant (Table 4).

The perception of alcohol consumption as a risk factor of oral cancer correlated with age (*P* < 0.001), education (*P* < 0.001), residence (*P* = 0.001), marital status (*P* = 0.004), and frequency of tooth brushing (*P* = 0.020) (Table 2). In the multivariate analysis, only age and residence showed statistical significance (Table 4).

### Awareness of the early signs of oral cancer

Approximately 40.0% of the respondents believed that the long-term unhealed ulcers in the mouth might be oral cancer, and 22.7% believed that white plaque in the mouth was the possible manifestation of oral cancer. Only 17.5% believed that red plaque was a possible manifestation of oral cancer. More than 60% of respondents had no knowledge of the early signs of oral cancer.

The perception of long-term unhealed ulcers as a risk factor was associated with age (*P* < 0.001), education (*P* < 0.001), residence (*P* < 0.001), income (*P* < 0.001), frequency of tooth brushing (*P* = 0.005), and mouth self-examination (*P* = 0.015) (Table 2). In multivariate analysis, age, residence, income, and mouth self-examination remained statistically significant (Table 5). Cognition of white plaque as an early sign of oral cancer correlated with gender (*P* = 0.031), age (*P* = 0.037), education (*P* = 0.001), residence (*P* = 0.003), income (*P* = 0.021), frequency of tooth brushing (*P* = 0.010), and frequency of mouth self-examination (*P* < 0.001) (Table 2). However, in the multivariate analysis, only residence and mouth self-examination were statistically significant (Table 5). Awareness of red plaque as a risk factor might be related to residence (*P* = 0.033), income (*P* = 0.024), frequency of tooth brushing (*P* = 0.007), and frequency of mouth self-examination (*P* = 0.001) (Table 2). In the multivariate analysis, only income and mouth self-examination were statistically significant (Table 5).

**Table 5:** Multiple Logistic regression analysis of factors associated with early signs of oral cancer

Variable	n (%)	OR	95% CI	*P*
*Ulcer*				
Age				
15–29 years	685 (42.3)	1		
30–44 years	387 (40.2)	0.834	0.702–0.992	**0.040**
45–59 years	103 (34.1)	0.781	0.587–1.040	0.091
≥ 60 years	47 (27.3)	0.653	0.428–0.995	**0.048**
Income				
< 20,000 Yuan	430 (37.9)	1		
20,000–40,000 Yuan	213 (35.7)	0.930	0.753–1.149	0.503
40,000–60,000 Yuan	193 (39.1)	1.094	0.875–1.369	0.430
> 60,000 Yuan	386 (46.5)	1.307	1.078–1.585	**0.006**
Mouth self-examination				
Never	170 (35.3)	1		
Nearly every day	199 (45.7)	1.397	1.063–1.837	**0.016**
Three or four times a week	417 (42.1)	1.238	0.981–1.563	0.072
Occasionally	436 (38.0)	1.066	0.848–1.339	0.585
*White plaques*				
Residence				
Downtown	122 (25.4)	1		
Suburbs	367 (23.9)	0.897	0.702–1.146	0.383
Outer suburbs	178 (19.3)	0.732	0.556–0.962	**0.025**
Rural	27 (23.1)	1.284	0.767–2.149	0.342
Mouth self-examination				
Never	91 (18.9)	1		
Nearly every day	121 (27.8)	1.535	1.117–2.110	**0.008**
Three or four times a week	260 (26.2)	1.470	1.115–1.937	**0.006**
Occasionally	222 (19.4)	1.017	0.770–1.343	0.904
*Red plaques*				
Income				
< 20,000 Yuan	178 (15.7)	1		
20,000–40,000 Yuan	92 (15.4)	0.966	0.731–1.277	0.809
40,000–60,000 Yuan	93 (18.9)	1.221	0.921–1.621	0.166
> 60,000 Yuan	173 (20.8)	1.280	1.006–1.629	**0.045**
Mouth self-examination				
Never	69 (14.3)	1		
Nearly every day	104 (23.9)	1.776	1.260–2.503	**0.001**
Three or four times a week	173 (17.5)	1.263	0.928–1.719	0.137
Occasionally	190 (16.6)	1.194	0.881–1.618	0.252

*P* value < 0.05 are shown in bold

### Awareness of prevention and treatment knowledge about oral cancer

Among the respondents, 18.7% believed that the most common age of oral cancer was between 40 and 60 years old, and 69.8% did not know. The gingival, lingual, and buccal mucosa were considered as the most common sites of oral cancer by 7.9%, 3.0%, and 2.7%, respectively, and 77.1% did not know. Among the respondents, 38.2% believed that oral cancer could be prevented and 60.3% did not know. In addition, 30.6% thought oral cancer could not be transmitted, and 64.8% did not know. Among the respondents, 46.2% thought we could reduce the risk of oral cancer by changing our lifestyle or habits, 52.7% did not know, and 0.7% said we could not. Moreover, 58.8% believed that early detection of oral cancer would improve the success rate of treatment, 40.4% had no clear idea.

When long-term non-healing ulcers, white plaques, or red plaques appeared in the oral cavity, most of the respondents (72.3%, 59.2%, and 59.3%, respectively) chose to go to the Department of Stomatology of general hospitals, while only a few (13.2%, 14.7%, and 15.1%, respectively) chose to visit a Stomatological Hospital. When there were lumps, persistent pain, bad breath, or loose teeth for unknown reasons, most of the respondents chose to go to the Department of Stomatology of the general hospital, while only a few chose to visit a Stomatological Hospital. The vast majority of those who choose to the Stomatological Hospital did not know at which department to register. More than 70% of the respondents were unaware of the therapeutic effect and cost of treatment; 62.3% were blind to the treatment methods; and 76.5% did not know the mortality rate of oral cancer.

## Discussion

Abnormalities in the mouth should be easy to notice, which can be screened by oral examination without computed tomography and other instruments. However, current data show that over half of patients with oral cancer were in an advanced stage when they visited the doctor, and many patients had a medical history of 2–6 months, which was mainly the result of lack of awareness of oral cancer. The purpose of this study was to understand the public knowledge of oral cancer. The convenience sampling method was used in this study.

The results of this study showed that public awareness of oral cancer is low, and about half had never even heard of oral cancer. In this study, the awareness rate of oral cancer was only 52.9%, far lower than other countries (84.2–95.6%) [4–7]. However, a few countries or regions reported a lower awareness rate of oral cancer among residents, such as 23.7% in Portugal [8], 30% in Tehran, Iran [9]. The awareness rate of oral cancer in India and Malaysia is closely related to the high incidence of oral cancer there. In Beijing, the incidence of oral cancer is lower.

Public awareness of risk factors associated with oral cancer is also weak. In this study, only 39.6% of respondents believed that smoking was a risk factor of oral cancer, which was far lower than other countries (54.5–92.4%) [6, 7, 10–14]. Only 37.2% identified tobacco chewing as a risk factor. There are many kinds of tobacco products and different ways of smoking tobacco. According to a survey conducted by Rogers et al. [14] in Liverpool, UK, in 2010, only 3% believed that tobacco chewing was one of the risk factors of oral cancer. As for tobacco use, 54.8–91.2% believed it was a risk factor of oral cancer [5, 6, 8, 11–13, 15, 16]. This may be related to the implementation of global anti-smoking actions in recent decades. Most people knew that smoking was harmful to health; however, most people believed that smokers were more likely to develop lung cancer. In fact, in addition to lung cancer, smokers are also more likely to develop throat and oral cancer [17].

In this study, only 25.5% thought that drinking alcohol was one of the risk factors of oral cancer, which was similar to that in Portugal (Oporto) [8] and the UK [14], higher than that in America (4.8%) [10], but less than that in most foreign reports (33.6–63.3%) [6, 7, 11–13, 15, 16]. 40.9% believed that betel nut chewing was one of the risk factors of oral cancer, which was significantly lower than the 54.5% reported in Malaysia in 2013 [7]. No reports on cognition of betel nut chewing were found in other countries or regions. This is related to the prevalence of betel nut chewing in Papua New Guinea, India, Sri Lanka, and some parts of Guangdong, Taiwan, and Hunan, where the prevalence of oral cancer is high [18]. While in Beijing, betel nut chewing is not prevalent. During the investigation, we found that most people did not know what areca nuts were. Most of those who were aware of areca nuts had never seen one, but had seen news reports about oral cancer after long term chewing of areca nuts.

In this study, 28.0% believed that age was a risk factor, which was lower than that reported in foreign studies (31.1–55%) [6, 11–13, 16]. Age is an important factor affecting the occurrence, treatment, and prognosis of most cancers. Exposure to potential carcinogens increases with age, and so does the likelihood of damage to the DNA of aging cells. The incidence of oral cancer increases with age. About 90% of oral cancer occurred in people over 40 years old, and about half occurred in people over 65 years old [19].

Public awareness of the early signs of oral cancer is also low. In this study, 40.0% believed that long-term non-healing ulcers in the mouth were an early manifestation or symptom of oral cancer, which was lower than that in other countries (57.3–90%) [5–7, 15, 20]. In this study, 17.5% believed that the red plaque was the early manifestation of oral cancer, and 22.7% believed that the white plaque was the early manifestation. In foreign studies, the recognition of the red plaque or white plaque as the early manifestations of oral cancer was slightly higher (39.8–58%) [6, 7, 15]. Most oral cancers develop from potentially malignant lesions that exist for a long time, especially in areas with a high incidence of oral cancer. Therefore, it is very important to strengthen public education on early warning signs of oral cancer to strengthen early diagnosis and treatment.

In this study, when asked where they would seek medical treatment for various oral problems, most respondents chose the Department of Stomatology of a general hospital, followed by a Stomatological Hospital. Patients with oral cancer were mainly seen in oral surgery, otolaryngology, and head and neck department. The early symptoms of some oral cancers are not typical and might be missed even by doctors specializing in oral and maxillofacial surgery. Gellrich et al. [21] conducted a retrospective study on 1761 patients, in which 1519 patients indicated the type of medical professionals who treated their initial symptoms: 40% of the patients were treated by dentists, 27% by family doctors, and 23% by oral and maxillofacial surgeons. Kowalski et al. [22] also obtained similar results in a prospective study on patients with oral cancer. Contrasting results have also been reported. Schnetler [23] found that family doctors diagnosed cancer and lymph node metastases earlier than dentists. Guggenheimer et al. [24] believed that because of the coexistence of systemic symptoms, the probability of receiving a diagnosis of suspected oral cancer from general health care providers was higher than that by dentists. People with tobacco and alcohol addiction are at high risk of oral cancer, as well as respiratory and digestive diseases, and cancers. Therefore, they are more likely to choose general practitioners rather than dentists if manifestations appear outside the oral region. Therefore, doctors who receive patients with suspected oral cancer also need to strengthen their oral cancer-related knowledge, in order to reduce the missed diagnosis of oral cancer and shorten the time of diagnosis delay.

A simple survey of personal oral hygiene habits was also conducted, in which 82.5% of the respondents reported brushing their teeth at least once a day in the morning and evening, but 1.5% of the respondents did not brush their teeth at all. About 46.6% had the habit of self-examination of their oral cavity. Generally, if oral problems occur, they should be easier to find; however, most of the clinical cases were locally advanced, largely because people do not understand the relevant early symptoms or manifestations, do not pay attention to them, and do not seek timely medical treatment. Self-examination has been used effectively to improve the early detection of breast cancer, and this strategy can also be applied to the early detection of oral cancer, but only if people's awareness level is improved.

Most of the previous literature and people generally believed that those with a higher education level and higher living standard have higher awareness of oral cancer [25–27]. According to the results of this survey, the main factors affecting people's awareness of oral cancer were age, living standards, and oral hygiene habits. Young people are relatively more exposed to a variety of new experiences and have a strong ability to learn and accept. People with higher living standards generally pay more attention to oral health and are more willing to go to the hospital for examination or treatment. Those who have good oral hygiene habits generally have good educational background and personal accomplishment. Therefore, it is very important to strengthen oral health education. Developing good oral hygiene habits can not only reduce the incidence of caries and periodontal disease, but also detect oral problems as soon as possible, so as to seek medical treatment early.

Many countries and regions have recognized the importance and necessity of publicity and education concerning oral cancer. Some organizations have made efforts to raise public awareness through brochures, television advertisements, and talk shows, etc.; and some studies have shown that people’s awareness of oral cancer can be influenced by those approaches [28, 29]. However, the data still shows that public awareness is low [10]. This study also investigated the channels through which people acquired oral health knowledge. Most of the respondents learned about oral cancer through TV programs (25.2%), phone news (20.3%), WeChat (13.0%), and talking with friends or neighbours (15.5%). Many studies have shown that the way people acquire knowledge about oral cancer is mainly through the media, and doctors only account for a small part. Therefore, in addition to the traditional paper media, we can use the fast, convenient, and rapid new media in modern society to publicize oral health knowledge to the public, such as TV programs, WeChat public numbers, short video applications, to publicize and educate the definition of oral cancer, related risk factors, early signs, self-examination methods, and oral health care methods. For medical workers, in the daily diagnosis and treatment process, they should also carry out oral health and oral cancer-related education according to the patient's own situation. For student groups, we can also set up special health lectures [30]. It is also important to raise the level of awareness of oral cancer among adolescents, considering that the mean age of individuals who start smoking and abusing alcohol is progressively decreasing.

## Conclusions

The results of the present study showed that the awareness rate of oral cancer in Beijing residents is low. Most residents know little or nothing about the risk factors and early symptoms of oral cancer, and some residents have not formed good personal oral hygiene habits. In this regard, targeted measures should be taken to improve the public's awareness of oral cancer and knowledge related to its prevention and treatment.

## Data Availability

All data generated or analysed during this study are included in this published article.

## References

[1] Bray F, Ferlay J, Soerjomataram I (2018). Global cancer statistics 2018: GLOBOCAN estimates of incidence and mortality worldwide for 36 cancers in 185 countries. CA Cancer J Clin.

[2] Chen W, Zheng R, Baade PD (2016). Cancer statistics in China, 2015. CA Cancer J Clin.

[3] Gao W, Guo CB (2008). Research status of delayed diagnosis in patients with oral squamous cell carcinoma. J Modern Stomatol.

[4] Grossmann S, Sales A, Reis D S, et al. Knowledge of oral cancer by a Brazilian population. J Cancer Educ. 2020; 1–6. 10.1007/s13187-020-01722-4 32124247

[5] West R, Alkhatib MN, McNeill A (2006). Awareness of mouth cancer in Great Britain. Br Dent J.

[6] Agrawal M, Pandey S, Jain S (2012). Oral cancer awareness of the general public in Gorakhpur city, India. Asian Pac J Cancer Prev.

[7] Ghani WMN, Doss JG, Jamaluddin M (2013). Oral cancer awareness and its determinants among a selected Malaysian population. Asian Pac J Cancer Prev.

[8] Monteiro LS, Warnakulasuriya S, Cadilhe S (2016). Oral cancer awareness and knowledge among residents in the Oporto city, Portugal. J Investig Clin Dent.

[9] Azimi S, Ghorbani Z, Ghasemi E (2019). Disparities in oral cancer awareness: a population survey in Tehran, Iran. J Cancer Educ.

[10] Luryi AL, Yarbrough WG, Niccolai LM (2014). Public awareness of head and neck cancers. JAMA Otolaryngol Head Neck Surg.

[11] Al-Maweri SA, Addas A, Tarakji B (2015). Public awareness and knowledge of oral cancer in Yemen. Asian Pac J Cancer Prev.

[12] Al-Maweri SA, Tarakji B, Alsalhani AB (2015). Oral cancer awareness of the general public in Saudi Arabia. Asian Pac J Cancer Prev.

[13] Eltayeb AS, Satti A, Sulieman AM (2017). Oral cancer awareness in Sudan: assessment of knowledge, attitude and treatment seeking behavior. Asian Pac J Cancer Prev.

[14] Rogers SN, Hunter R, Lowe D (2011). Awareness of oral cancer in the Mersey region. Br J Oral Maxillofac Surg.

[15] Monteiro LS, Salazar F, Pacheco J (2012). Oral cancer awareness and knowledge in the city of Valongo, Portugal. Int J Dent.

[16] Hertrampf K, Wenz H-J, Koller M (2012). Public awareness about prevention and early detection of oral cancer: a population-based study in Northern Germany. J Cranio Maxillofac Surg.

[17] Warnakulasuriya S, Dietrich T, Bornstein MM (2010). Oral health risks of tobacco use and effects of cessation. Int J Dent.

[18] Liu ST, Wu JF, Zheng RS (2013). Incidence and mortality of oral cavity and pharyngeal cancer in China, 2009. Chin J Prev Med.

[19] Shiboski CH, Shiboski SC, Silverman S (2000). Trends in oral cancer rates in the United States, 1973–1996. Commun Dent Oral Epidemiol.

[20] Kumar A, Jha R, Kumar N (2017). A study of the awareness of oral cancer and its Associated Risk Factors amongst O.P.D. Attendees at a Teaching Hospital of Bhubaneswar, India. Int Arch BioMed Clin Res.

[21] Gellrich NC, Suarez-Cunqueiro M, Bremerich A (2003). Characteristics of oral cancer in a central European population: defining the dentist's role. J Am Dent Assoc.

[22] Kowalski L (1994). Lateness of diagnosis of oral and oropharyngeal carcinoma: factors related to the tumour, the patient and health professionals. Eur J Cancer B Oral Oncol.

[23] Schnetler JFC (1992). Oral cancer diagnosis and delays in referral. Br J Oral Maxillofac Surg.

[24] Guggenheimer J, Weissfeld JL, Kroboth FJ (1993). Who has the opportunity to screen for oral cancer?. Cancer Causes Control.

[25] Azimi S, Ghorbani Z, Ghasemi E (2020). Does socioeconomic status influence oral cancer awareness? The role of public education. East Mediterr Health J..

[26] Kawecki MM, Nedeva IR, Iloya J (2019). Mouth cancer awareness in general population: results from Grampian Region of Scotland, United Kingdom. J Oral Maxillofac Res.

[27] Grossmann S, Sales A, Reis DS (2020). Knowledge of oral cancer by a Brazilian population. J Cancer Educ.

[28] Eadie D, Mackintosh AM, Macaskill S (2009). Development and evaluation of an early detection intervention for mouth cancer using a mass media approach. Br J Cancer.

[29] Siddique I, Mitchell DA (2013). The impact of a community-based health education program on oral cancer risk factor awareness among a Gujarati community. Br Dent J.

[30] Rupel K, Ottaviani G (2019). Campaign to increase awareness of oral cancer risk factors among preadolescents. J Cancer Educ..

